# Implementation of recommendations on the check of risk factors for cardiovascular diseases in patients undergoing coronary re-interventions

**DOI:** 10.1007/s11845-023-03285-x

**Published:** 2023-01-27

**Authors:** Marcin Walukiewicz, Daniel Rogowicz, Łukasz Wołowiec, Małgorzata Chudzińska, Anna Sinkiewicz, Grzegorz Grześk

**Affiliations:** 1 Department of Cardiology and Clinical Pharmacology, Faculty of Health Sciences, Ludwik Rydygier Collegium Medicum in Bydgoszcz, Nicolaus Copernicus University in Toruń, Ujejskiego 75 Street, Bydgoszcz, 85-168 Poland; 2 Department of Nutrition and Dietetics, Faculty of Health Sciences, Ludwik Rydygier Collegium Medicum in Bydgoszcz, Nicolaus Copernicus University in Toruń, Dębowa Street, Bydgoszcz, 85-626 Poland; 3 Department of Otolaryngology, Audiology and Phoniatrics, Faculty of Health Sciences, Ludwik Rydygier Collegium Medicum in Bydgoszcz, Nicolaus Copernicus University in Toruń, Ujejskiego 75 Street, Bydgoszcz, 85-168 Poland

**Keywords:** Cardiovascular disease, Cholesterol, Coronary angioplasty, Repeated revascularization, Secondary preventions

## Abstract

**Method:**

The study involved 905 patients after coronary interventions, qualified for invasive diagnosis due to symptomatic coronary disease.

**Aim:**

The aim of this study was to check the implementation of recommendations on the control of risk factors for cardiovascular diseases in patients undergoing re-interventions.

**Results:**

Compared to elderly persons, younger people more often increased their physical activity (62 vs. 65 years, *p* = 0.009), stopped smoking (61 vs. 65 years, *p* < 0.001) and reduced alcohol consumption (62 vs. 65 years, *p* = 0.001). People with secondary and higher education increased their physical activity more often than those with primary education (51%, 31% vs. 23%, *p* = 0.006). Men more often than women decided to limit their alcohol consumption (48% vs. 37%, *p* = 0.007). Patients with a history of acute coronary syndrome were more likely to quit smoking and reduce their alcohol consumption than those without such a history (47% vs. 37%, *p* = 0.003 and 42% vs. 34%, *p* = 0.020, respectively). Only 2% of the subjects achieved the recommended LDL cholesterol values. Forty-eight percent were qualified for reinvasive procedures on the coronary arteries. Less than half of the patients undertook health-promoting behaviors that required modification of existing habits.

**Conclusion:**

Age, gender, and education level influence pro-health behaviors. The majority of patients do not achieve the levels of LDL cholesterol and triglycerides consistent with the ESC guidelines in the secondary prevention of coronary disease. Inadequate check of risk factors may result in faster disease progression and coronary re-interventions.

## Introduction

The growing number of patients hospitalized for cardiovascular reasons in Poland and Europe every year is a huge challenge for the modern healthcare system. Despite significant changes in recent years in the field of pharmacotherapy, prevention and invasive treatment, cardiovascular diseases (CVD) are a major problem not only economically, but also socially. In addition to diagnostics, treatment, rehabilitation, and care at the highest level, prevention and health promotion seem to be the key issues that can effectively contribute to increasing the population's awareness of risk factors for developing cardiovascular disease [1].

Modifiable risk factors (factors that can be changed, for example, smoking, physical inactivity, diet, obesity, high blood pressure) predisposing to the development of cardiovascular diseases have been known for many years. However, their correct identification and elimination are very difficult for many patients, which is strongly related to the level of their knowledge and socio-economic status. The most important thing in the early stages of atherosclerosis is to stop its further progression and to prevent serious consequences as well as adverse cardiovascular events, such as heart attack, ischemic stroke, heart failure, or death. Taking appropriate actions, estimating the risk as well as proper prevention and prophylaxis turn out to be essential for reducing the incidence and mortality from cardiovascular causes [1]. In the case of CVD, the priority seems to be prevention, including lifestyle changes and adherence to pharmacotherapy recommendations [2–6]. The decisive risk factors responsible for the increase in mortality due to cardiovascular events are smoking, excessively high cholesterol values, hypertension, and diabetes [1]. The fundamental aspects in achieving the intended therapeutic goal are the education of both the patients and their families, who also actively participate in the treatment process, and personalized pharmacotherapy [7, 8]. It is worth mentioning that the method of therapeutic management is closely related to patients’ condition, the level of their awareness and involvement in the process of convalescence and recovery [1].

Atherosclerosis is the main cause of the development of cardiovascular diseases. Epidemiological data are alarming, putting CVD at the top of the causes of death in both Poland and Europe. Based on the European report, which classifies and statistically presents the causes of population deaths, a significant problem has been identified—the dominance of CVD (45% and 39% of all deaths in females and males) compared to other diseases such as cancer or traffic accidents. CVD is the leading cause of mortality, responsible for 2.2 million deaths in females and just over 1.9 million deaths in males. In 2015, the European Heart Network estimated that CVD cost the European Union economy €210 billion [9].

The aim of our study was to assess the awareness and knowledge of patients as well as the practical implementation of a therapeutic plan that includes the elimination of risk factors for the progression of cardiovascular diseases in patients undergoing coronary re-interventions.

## Materials and methods

### Ethics statement, material, and protocol

This was a single-center study, conducted with the consent of the Bioethics Committee No. KB 198/2008 at the Department of Cardiology and Clinical Pharmacology of the Dr. Jan Biziel University Hospital No. 2 in Bydgoszcz. The duration of the study was 6 years.

Patients with coronary artery disease (CAD) undergoing repeated invasive diagnostics were enrolled. The study included a group of 1000 people hospitalized in the Department of Cardiology and Clinical Pharmacology of the Dr. Jan Biziel University Hospital No. 2 in Bydgoszcz. The mean follow-up time from the first coronary intervention was 3 years and 8 months.

Detailed data was collected based on a previously prepared questionnaire, which was approved by the local Bioethics Committee. For the purposes of the analysis, 905 completed questionnaires were collected. The survey questionnaire has been divided into 3 main sections including the following:

I. Socio-economic indicators such as gender, age, place of residence, professional activity, and education.
II. Basic medical data: height and body weight, abdominal circumference, systolic, and diastolic blood pressure values, heart rate, comorbidities, past procedures, treatment used, the frequency of cholesterol value control as well as the previous and current values of serum levels of glucose, LDL and TG.
III. Introduced pro-health behaviors: diet modification, smoking cessation, increasing physical activity, the control of lipid profile and blood pressure, and weight reduction.

The survey questionnaire contained both closed questions with ready-made answer forms as well as open-ended questions. The survey was carried out routinely, simultaneously with the measurements of vital signs and indices concerning body weight, height, and abdominal circumference by qualified nursing and medical personnel.

### Statistical analysis

The results of the survey were compiled using Microsoft Office Excel. Statistical analyzes were performed using the STATISTICA 12.5 PL software (Statsoft, Kraków, Poland). Data in the nature of quantitative variables were presented in the form of the mean (M) and standard deviation (SD), as well as the minimum (Min), maximum (Max), 10th and 90th percentiles and the median (Me). Data in the nature of qualitative (categorized) variables were described by comparing the relative number of cases (*N*) and their percentage share (%) in the studied group.

### Study limitations

This is a single-center study, which included heterogeneous patients with the symptomatic chronic coronary syndrome (chronic coronary syndrome treated conservatively, patients with a history of ACS, patients after CABG and PCI) who were eligible for follow-up coronary angiography. The data were gathered from questionnaires collected. Unfortunately, we could not assess the association between hypercholesterolemia management and the risk of cardiovascular events.

## Results

The general characteristics of the group are presented in Table 1. The age range in the studied group ranged from 35 to 88 years. The mean age of the subjects was 64 ± 9.7 years and 80% of the subjects were between 52 and 77 years of age. Among 905 patients studied, 356 were women, which accounted for 39% of the respondents, and 549 men, which corresponded to 61% of the subjects. The vast majority of patients lived in cities with populations of less than 50000 (39%, 354 people), had vocational education (41%, 354 people), and were retired (53%, 478 people).

**Table 1:** Characteristic of study group

	**Study group (*n* = 905)**
Age (years)*	64.2 ± 9.7
Men	61%
Women	39%
BMI (kg/m^**2**^)*	28.6 ± 4.45
BMI = 18.5–24.9 kg/m^**2**^	21%
BMI = 25–29.9 kg/m^**2**^	44%
BMI > 30 kg/m^**2**^	35%
Abdominal circumference (cm)*	101.3 ± 12.4
Place of residence	
Village	30%
City < 50 thous. residents	39%
City 50–100 thous. residents	6%
City > 100 thous. residents	25%
Education	
Primary	23%
Vocational	41%
Secondary	6%
Higher	30%
Professional activity	
Professionally active	18%
Retired	53%
Pensioner	29%
Comorbid disease	
ACS	75%
STEMI	30%
NSTEMI	27%
UA	18%
AHT	80%
DMT2	32%
CKD	9%
Peripheral arterial disease	8%
Stroke	7%
Carotid artery disease	7%
Intervention	
Coronarography	100%
PTCA	66%
POBA	9%
CABG	17%
Applied drugs	
ASA	98%
Clopidogrel	65%
Statin	95%
Βeta-blocker	93%
ACEI	87%
ARB	7%
Checking cholesterol	
Every 3 months	32%
Every 6 months	29%
Once a year	22%
Not at all	17%
The cause of checking cholesterol less frequently than every 3 months	
Lack of knowledge	25%
Lack of doctor’s consent	33%
No need	10%
Pro-health behaviors	
Check of blood pressure	94%
Check of lipid profile values	82%
Modification of diet	74%
Cessation of smoking (if applicable)	43%
Reduction in alcohol consumption	39%
Weight reduction	34%
Increase in physical activity	30%

The results in the tables are presented as follows: *means ± standard deviation

*ACEi* angiotensin-converting-enzyme inhibitors, *ACS* past acute coronary syndrome, *AHT* atrial hypertension, *ARB* angiotensin II receptor blockers, *ASA* acetylsalicylic acid, *BMI* body mass index, *CABG* coronary artery bypass graft, *CKD* chronic kidney disease, *DMT2* diabetes mellitus type 2, *NSTEMI* non-ST-segment elevation myocardial infarction, *POBA* percutaneous old balloon angioplasty, *PTCA* percutaneous transluminal coronary angioplasty, *STEMI* ST-segment elevation myocardial infarction, *UA* unstable angina

In the studied group, only 290 people (32%) replied that they had their blood cholesterol levels checked every 3 months. Check of cholesterol value concerned the period between the first and subsequent coronary interventions.

In addition to the question about the frequency of cholesterol checks, respondents were asked about the reason for checking at such intervals. Subjects, whose blood cholesterol results were performed less frequently than once every 3 months (this was the case for as many as 68% of the subjects), indicated various reasons, namely:

- 37% of persons pointed to a lack of knowledge about the frequency with which blood biochemistry tests should be performed;
- as many as 48% of respondents indicated a lack of consent from their primary care physician to issue a referral for laboratory tests;
- the remaining 15% of persons indicated no need to check this parameter.

With regard to CVD, which were the cause of the first coronary intervention, as many as 75% of the subjects experienced acute coronary syndrome in the studied group. Thirty percent of the subjects (271 persons) experienced ST-segment elevation myocardial infarction, 27% (242 persons) had non-ST-segment elevation myocardial infarction, while 18% of the subjects (166 persons) were treated for unstable angina. In the studied group, the most common comorbidity was arterial hypertension and it concerned as much as 80% of the respondents (722 persons). The next most common disease was diabetes, which affected 32% of the subjects (294 persons). The other comorbidities, occurring less frequently, were as follows: chronic kidney disease, which was reported in 9% of the subjects, peripheral arterial disease occurring in 8%, carotid artery disease in 7% and a history of stroke, which occurred in 7% of the studied persons.

In the studied group, after the first coronary angiography, 599 persons (66% of the respondents) underwent angioplasty with stent implantation, 152 persons (17%) underwent a coronary aortic bypass surgery, and 78 persons (9%) reported balloon angioplasty in a medical history.

The most frequently used drug was acetylsalicylic acid—98% of the subjects, statins were taken by 95% of patients, 93% of patients had β-blockers included in the therapy. Angiotensin-converting enzyme inhibitors were taken by 87% of the subjects, and clopidogrel by 65%.

Among the pro-health behaviors used, the largest number of persons declared blood pressure control (854, which constituted 94% of the subjects) and 754 persons (82%)—check of the lipid profile values. A total of 393 subjects (43%) stopped smoking, 357 (39%) limited their alcohol consumption, 309 (34%) reduced their body weight, and 257 (30%) increased their physical activity.

The Mann–Whitney *U* test was used to analyze the differences between the age groups of subjects participating in the survey and the type of pro-health behaviors undertaken by these persons. The results of this analysis are presented in Table 2. Statistically significant differences were observed for the following variables: increased physical activity (*p* = 0.009), cessation of smoking (*p* < 0.001), and reduced alcohol consumption (*p* = 0.001).

**Table 2:** Pro-health behaviors undertaken depending on age

**Pro-health behaviors**		***N***	**Me**	**IQR**	***Z***	***p***
Modification of diet	Yes	674	63.0	15.0	−0.16	0.837
	No	231	64.0	14.0		
Increase in physical activity	Yes	267	62.0	13.0	2.34	0.009
	No	638	65.0	16.0		
Check of lipid profile values	Yes	739	70.0	15.0	0.34	0.345
	No	160	70.0	18.0		
Check of blood pressure	Yes	848	70.0	16.0	− 0.85	0.396
	No	51	72.0	15.0		
Cessation of smoking	Yes	389	61.0	16.0	4.56	< 0.001
	No	510	65.0	15.0		
Reduction in alcohol consumption	Yes	354	62.0	16.0	3.19	0.001
	No	545	65.0	14.0		
Weight reduction	Yes	306	70.0	16.0	0.19	0.846
	No	593	70.0	16.0		

*N* number, *Me* median, *IQR* interquartile range, *Z* test statistic value, *p* statistical significance level

In order to assess the differences in pro-health behaviors undertaken depending on gender, a chi-square test was performed. The results of the analysis are presented in Table 3. The only statistically significant difference in the pro-health behaviors between the male and female groups was the reduction in alcohol consumption in the male population. This was the case for 48% of men compared to 37% of women (*p* = 0.007). There were no statistically significant differences between other pro-health behaviors in the studied group.

**Table 3:** Differences in pro-health behaviors undertaken by women and men

**Pro-health behaviors**		**Women**		**Men**		**chi**	***p***
		***N***	**%**	***N***	**%**		
Modification of diet	Yes	269	76%	405	74%	0.36	0.541
	No	87	24%	144	26%		
Increase in physical activity	Yes	116	33%	356	65%	0.92	0.761
	No	240	67%	193	35%		
Check of lipid profile values	Yes	297	83%	448	82%	0.49	0.482
	No	59	17%	101	18%		
Check of blood pressure	Yes	300	84%	447	81%	0.32	0.571
	No	62	17%	102	19%		
Cessation of smoking	Yes	341	96%	513	93%	2.23	135
	No	15	4%	36	7%		
Reduction in alcohol consumption	Yes	130	37%	263	48%	11.4	0.007
	No	226	63%	286	52%		
Weight reduction	Yes	116	33%	193	35%	0.63	0.426
	No	240	67%	356	65%		

*N* number, *p* statistical significance level, *chi* chi-square test

The relationship between education and the applied pro-health behaviors was analyzed and the results of the analysis are presented in Table 4. In the studied group, significant differences were observed between the choice of pro-health behaviors and the level of education. Statistically significant results were obtained for the variables: an increase in physical activity (*p* = 0.006) and a reduction in alcohol consumption (*p* = 0.044). Persons with secondary education significantly more often increased their physical activity (51%) compared to the rest. Unfortunately, this pro-health change was observed only in 31% of persons with higher education, in 28% with vocational education, and only in 23% with primary education. Limiting alcohol consumption was declared by the largest number of persons with secondary education—51%, while in the other groups, it was respectively 42% with vocational education, 38% with higher education and 33% with primary education.

**Table 4:** Pro-health measures taken depending on the education level

**Pro-health behaviors**		**Primary education**		**Vocational education**		**Secondary education**		**Higher education**		**chi**	***p***
		*N*	%	*N*	%	*N*	%	*N*	%		
Modification of diet	Yes	140	69%	276	75%	48	84%	210	77%	7.19	0.066
	No	64	31%	94	25%	9	16%	64	23%		
Increase in physical activity	Yes	47	23%	105	28%	29	51%	86	31%	17.31	0.006
	No	154	75%	265	72%	28	49%	188	69%		
Check of lipid profile values	Yes	161	79%	301	81%	52	91%	231	84%	5.71	0.125
	No	43	21%	69	19%	5	9%	43	16%		
Check of blood pressure	Yes	189	93%	352	95%	54	95%	259	95%	1.57	0.665
	No	15	7%	18	5%	3	5%	15	5%		
Cessation of smoking	Yes	86	42%	167	45%	26	46%	114	42%	1.05	0.788
	No	118	58%	203	55%	31	54%	160	58%		
Reduction in alcohol consumption	Yes	68	33%	157	42%	29	51%	103	38%	0.81	0.044
	No	136	67%	213	58%	28	49%	171	62%		
Weight reduction	Yes	66	32%	121	33%	25	44%	97	35%	3.22	0.359
	No	138	68%	249	67%	32	56%	177	65%		

*N* number, *p* statistical significance level, *chi* chi-square test

A chi-square test was performed in order to assess the differences between the applied pro-health behaviors depending on professional activity. Table 5 shows the results of the analysis. The largest number of persons who increased their physical activity (38%) was recorded in the group of professionally active subjects. In the case of retired subjects and disability pensioners, only 27% and 29%, respectively, decided to change their physical activity. Most persons on disability pensions quit smoking—53%. Among professionally active people, the choice of introducing this pro-health change was made by 44% of the respondents. The smallest number of people who quit smoking (38%) was in the group of retired persons. The consumption of alcohol products was reduced to the greatest extent (45%) among disability pensioners and in 43% of professionally active people. The lowest reduction in alcohol consumption was recorded in 32% of retired subjects.

**Table 5:** Effect of professional activity on undertaken pro-health behaviors

**Pro-health behaviors**		**Professionally active people**		**Retired people**		**People on disability pensions**		**chi**	***p***
		*N*	%	*N*	%	*N*	%		
Modification of diet	Yes	131	80%	348	73%	195	74%	3.14	0.207
	No	33	20%	129	27%	69	26%		
Increase in physical activity	Yes	63	38%	127	27%	77	29%	8.17	0.019
	No	101	62%	350	73%	187	71%		
Check of lipid profile values	Yes	135	82%	392	82%	218	83%	0.18	0.991
	No	29	18%	85	18%	46	17%		
Check of blood pressure	Yes	157	96%	447	94%	250	95%	1.01	0.602
	No	7	4%	30	6%	14	5%		
Cessation of smoking	Yes	72	44%	180	38%	141	53%	17.01	< 0.001
	No	92	56%	297	62%	123	47%		
Reduction in alcohol consumption	Yes	69	42%	169	35%	119	45%	7.19	0.027
	No	95	58%	308	65%	145	55%		
Weight reduction	Yes	63	38%	160	34%	86	33%	1.69	0.428
	No	101	62%	317	66%	178	67%		

*N* number, *p* statistical significance level, *chi* chi-square test

The study also assessed the effect of past acute coronary syndrome on the implementation of pro-health behaviors. The results obtained in the course of the analysis are presented in Table 6. The results showed statistically significant differences in smoking cessation (*p* = 0.003) and reduction in alcohol consumption (*p* = 0.020), and these behaviors were strongly associated with a history of ACS. In the group of people who had ACS, 47% declared quitting smoking, while in the group of people who did not have ACS, these changes were introduced by 37% of them. Forty-two percent of people after ACS reduced their alcohol intake, compared to 34% of subjects without a history of ACS.

**Table 6:** Effect of past ACS on the choice of pro-health behaviors

**Pro-health behaviors**		**ACS**		**No ACS**		**chi**	***p***
		*N*	%	*N*	%		
Modification of diet	Yes	443	75%	84	27%	0.33	0.534
	No	147	25%	231	73%		
Increase in physical activity	Yes	172	29%	95	30%	0.09	0.751
	No	418	71%	220	70%		
Check of lipid profile values	Yes	488	83%	257	82%	0.18	0.672
	No	102	17%	58	18%		
Check of blood pressure	Yes	562	95%	292	93%	2.52	0.112
	No	28	5%	23	7%		
Cessation of smoking	Yes	277	47%	116	37%	8.56	0.003
	No	313	53%	199	63%		
Reduction in alcohol consumption	Yes	249	42%	108	34%	5.38	0.020
	No	341	58%	207	66%		
Weight reduction	Yes	206	35%	103	33%	0.45	0.502
	No	384	65%	212	67%		

*N* number, *ACS* acute coronary syndrome, *p* statistical significance level, *chi* chi-square test

The one-way ANOVA test with repeated results was used to check correlations between the results of biochemical laboratory parameters such as glucose, LDL and TG, which were determined during the first coronary intervention and during the current hospitalization. The results obtained in the course of the above analysis are presented in Table 7. Current and previous LDL cholesterol values were obtained from 862 subjects. The previous mean value of LDL concentration was 135.36 ± 43.701267 (mg/dL), while the mean current value of this parameter was 114.02 ± 35.5617 (mg/dL). The mean current LDL cholesterol was approximately 20 mg/dL lower compared to the value during the first coronary intervention. Data on triglyceride concentration values were obtained from 849 subjects. The previous mean value of TG concentration was 164.07 ± 76.169 (mg/dL), while the mean current value of this parameter was 150.02 ± 76.416 (mg/dL). As in the case of LDL cholesterol, a downward trend was also noted for TG. The mean measurement results of this parameter were lower in the study population by approximately 14 mg/dL.

**Table 7:** Comparison of the current and previous concentration values of biochemical parameters: glucose, LDL, and TG in the studied group

**Biochemical parameters**				*F*	*p*
		**Previous values**	**Current values**		
Glucose(mg/dl)	N	866	866	0.16	0.900
	M	111.24	111.42		
	SD	38.237	33.297		
LDL(mg/dl)	N	862	862	227.10	< 0.001
	M	135.36	114.02		
	SD	43.701	35.561		
TG(mg/dl)	N	849	849	33.69	< 0.001
	M	164.07	150.126		
	SD	76.169	76.416		

*N* number, *M* mean, *SD* standard deviation, *LDL* low-density lipoproteins, *TG* triglycerides, *F* Fisher’s statistic value, *p* statistical significance level

**Fig. 1:**
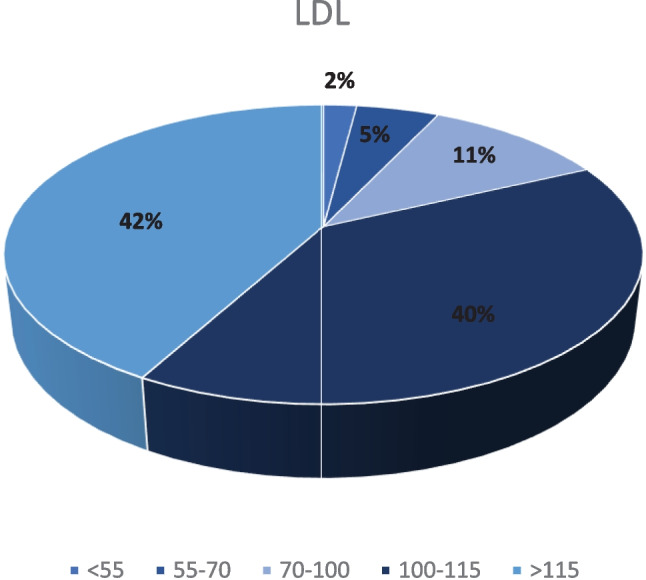
Pie chart showing the division of the subjects according to the current level of LDL cholesterol

Despite the observed downward trend in LDL cholesterol concentration, only 2% of the subjects achieved the target values in the secondary prevention of coronary disease. LDL level < 70 mg/dL was reached by 7% of the observed patients. The results depending on the achieved LDL cholesterol concentration in the studied group are presented in Fig. 1. Blood pressure below 140/90 mmHg was achieved by 77% of the subjects.

**Fig. 2:**
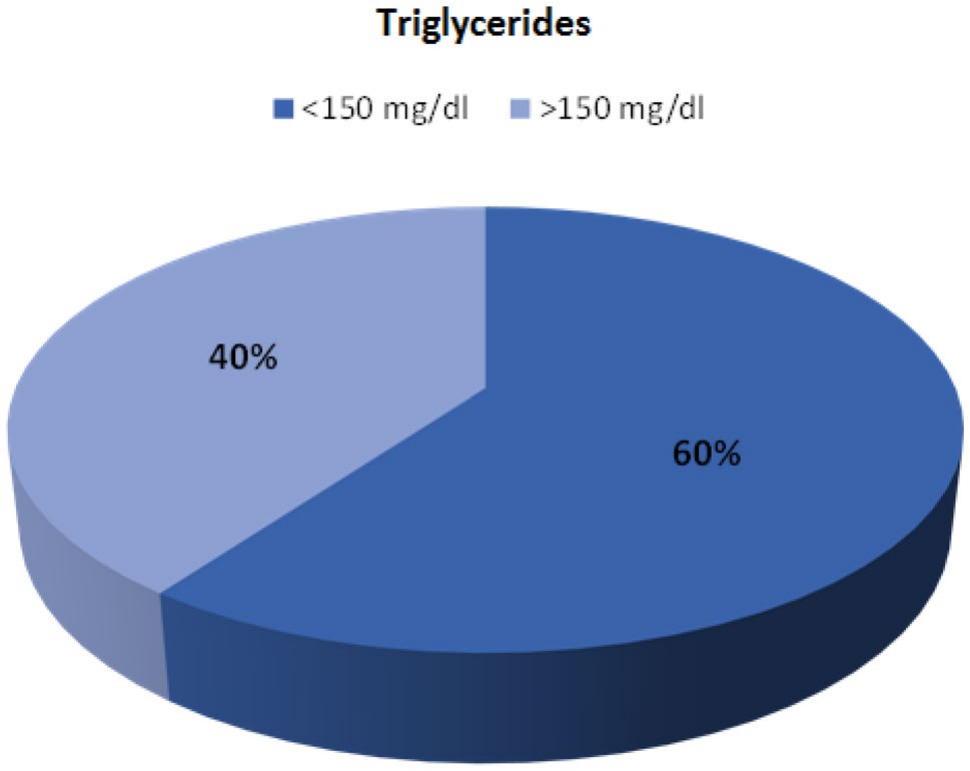
Pie chart showing the division of the subjects according to the current level of triglycerides

With regard to triglyceride concentration, the target values were reached by 60% of the respondents. The results depending on the achieved triglyceride concentration in the studied group are presented in Fig. 2.

**Fig. 3:**
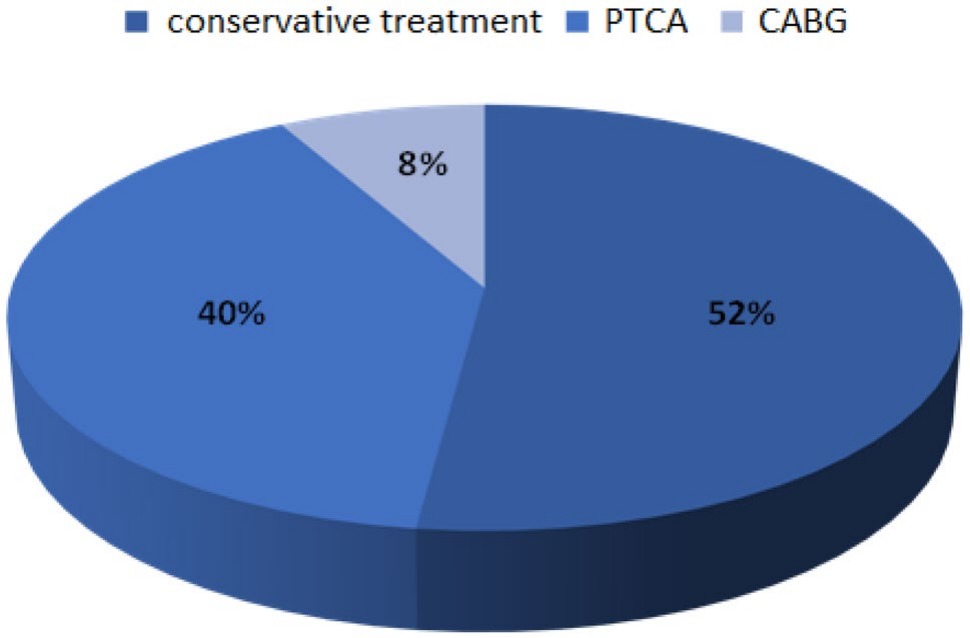
Pie chart showing the division of subjects according to the type of treatment used after re-coronary angiography. PTCA percutaneous transluminal coronary angioplasty, CABG coronary artery bypass graft

Patients after coronary angiography were divided according to the applied method of treatment. The results are shown in Fig. 3. Conservative treatment was applied in 52% of patients, 40% underwent re-angioplasty and 8% were qualified for cardiac surgery.

## Discussion

Cardiovascular diseases, especially CAD, are one of the main causes of morbidity, disability, and premature deaths in Poland and Europe [1, 9]. As it follows from the INTERHEART study, modifiable risk factors are responsible for 90% of heart attacks [10]. According to a WHO report, 75% of deaths from cardiovascular disease can be prevented by lifestyle changes [11]. Prevention of these diseases at both the individual and population levels is therefore a major challenge for healthcare systems. In most cases, however, they are mainly focused on intensive medical care, pharmacological treatment, or the introduction of still newer medical technologies, while the modification of cardiovascular risk factors, i.e., broadly understood prevention, despite specific recommendations, is left to patients as a matter of their own choice [12]. Thus, insufficient knowledge and awareness of risk factors are the reasons for the ineffectiveness of both primary and secondary prevention [12]. This situation is reflected in the analysis of the results of epidemiological studies NATPOL 11 [13], NATPOL PLUS [14], 3ST-POL [15], WOBASZ (2003–2005), WOBASZ SENIOR [16], and WOBASZ II (2013–2015) [17], whose aim was to analyze the prevalence of risk factors for the development of cardiovascular diseases and dyslipidemia. The two-fold increase in the frequency of cholesterol level check observed in 2005–2015, both in people without risk factors and in the group of secondary prevention, should result in improved treatment effects [18]. In the EUROASPIRE II, III, and IV studies during the follow-up in 1999–2013, the percentage of patients with established CAD who achieved the secondary prevention goal of LDL < 1.8 mmol/L (< 70 mg/dL) was 6.1%, 20.9%, and 25.6%, respectively. The observed patients took lipid-lowering drugs (statins, fibrates, bile acid sequestrants, nicotinic acid and its derivates, cholesterol absorption inhibitors) in the EUROASPIRE surveys II, III, and IV at the following rates 60.5%, 90.1%, and 89.8% [19]. In the EUROASPIRE surveys V in 2016–2017, LDL < 1.8 mmol/L (< 70 mg/dL) level was achieved by about 30% of the observed subjects, 92% of whom declared regular intake of lipid-lowering drugs [20].The observational Dyslipidemia International Study II (DYSIS II) in 2012–2014 evaluated the achievement of LDL targets in patients with stable CAD and acute coronary syndrome in relation to effective lipid-lowering therapy, which was defined as monotherapy, non-statin monotherapy, statin plus ezetimibe, and statin plus non-statin medication (fibrates, omega-3 fatty acids, and nicotinic acid). The percentage of patients with stable CHD who achieved LDL < 1.8 mmol/L (< 70 mg/dL) was 23.2% for the LLT group and 2.9% for the no-LLT group. The percentage of patients with ACS who achieved LDL < 1.8 mmol/L (< 70 mg/dL) was 23.8% for the LLT group and 3.6% for the no-LLT group [21]. In the 2017–2018 DAVINCI study, patients with established atherosclerotic cardiovascular disease (ASCVD, defined as coronary disease, cerebral disease, peripheral disease and other evidence of atherosclerosis or other manifestation of vascular disease) achieved target LDL < 1.8 mmol/L (< 70 mg/dL) in 39% and LDL < 1.4 mmol/L (< 55 mg/dL) in 18%. However, patients with coronary diseases from ASCVD achieved LDL < 1.8 mmol/L (< 70 mg/dL) in 44% and LDL < 1.4 mmol/L (< 55 mg/dL) in 20%. The use of statin and ezetimibe therapy (9% of the observed subjects) allowed for achieving LDL < 1.8 mmol/L (< 70 mg/dL) in 54%, and LDL < 1.4 mmol/L (< 55 mg/dL) in 21% of the subjects. Combining PCSK9 with ezetimibe plus statin (1% of the subjects observed), LDL < 1.8 mmol/L (< 70 mg/dL) stability was achieved in 67% and LDL < 1.4 mmol/L (< 55 mg/dL) in 58% of the patients [22]. Unfortunately, despite this and the widely used statin therapy, the percentage of patients with hypercholesterolemia who achieved the treatment goal in Poland increased only from 3 to 6% over the decade [17]. In Poland, the responsibility for implementing the principles of prevention rests mainly with general practitioners. According to the analysis of the WOBASZ II study, 80% of the respondents had contact with a general practitioner at least once a year and 2/3 of them declared that they had received an advice on prophylaxis [18]. On the other hand, however, every third respondent had never received any advice on prophylaxis, and what is more, they had not had blood pressure or lipid levels measured. In the subgroup of people from secondary prevention, only 70% of the respondents (75% of men and 65% of women) checked their cholesterol levels, which is comparable to the results of the present study in which it was 82%. In our study, every third respondent had cholesterol levels checked every 3 months, every fifth respondent yearly, and 17% had no cholesterol checks at all. Among people controlling cholesterol levels less frequently than every 3 months, as many as 48% of the respondents indicated the lack of consent from their physician to perform a blood test, 37% declared that they had no knowledge about the need to check lipid parameters, and 15% were aware of this but did not consider it necessary. A decrease in the mean concentration of LDL cholesterol by 20 mg/dL to the value of 114.02 mg/dL was observed among the respondents, but only 2% of them achieved the currently recommended level of lipid concentration (LDL < 55 mg/dL) and 7% reached LDL < 70 mg/dL. This is much less compared to the study by Jankowski et al., where in a group of 595 patients after acute coronary syndrome or myocardial revascularization, the target LDL cholesterol concentration (< 70 mg/dL) was reached by 27% of the subjects [23]. It is difficult to assess what may be the reason for such a difference, taking into account the fact that there are no significant differences in both groups in terms of age (62.8 vs. 64.2), sex (M 66.7%; F 33.3% vs. M 61%; K 39%), BMI > 25 kg/m^2^ (80.4% vs. 79%) or the declared diet modification (80% vs. 74%). The frequency of lipid-lowering therapy in the study by Jankowski et al. on discharge from the hospital was 94%, and in a year and a half of follow-up, it was only 82%, similar to the Poznań registry in 3500 patients hospitalized due to STEMI[24]. In our study, the percentage of patients using statins during the subsequent coronary intervention (on average after 3 years and 8 months of follow-up) was higher and amounted to 95%. The difference in the achieved target lipid values may result from a longer follow-up period in our study and possibly, the associated less intensive check of patients with the time elapsing from a coronary event, both by GPs and in specialist clinics. In view of the above results, the catastrophically low effectiveness of dyslipidemia treatment in Poland should be one of the main goals of CVD prevention. In the study group of patients, significantly better control of hypertension was observed than in the study by Jankowski et al. [23]. Normal arterial pressure (< 140/90 mm Hg) was found in 77% of patients, compared to 53% in the Krakow population. This may result, inter alia, from the frequency of used beta-blockers (93% vs. 82%), angiotensin-converting enzyme inhibitors/sartans (94% vs. 78%).

The level of knowledge and awareness of risk factors for CVD, as shown in the analysis of WOBASZ, is closely related to age and education level [18]. In our study, also younger and better-educated people significantly more often reduced their alcohol consumption, increased physical activity, and quit smoking. Additionally, the influence on the control of risk factors in our population was related to gender, as men reduced alcohol consumption more often than women (48% vs. 37%, *p* = 0.007). Noteworthy is a much smaller percentage of pro-health behaviors that required personal constant effort and resignation from previous habits. Regular blood pressure check (94% of patients) or cholesterol level measurements (82%) was declared by significantly more respondents than quitting smoking (43%), reducing alcohol consumption (39%), decreasing body weight (34%) or increasing physical activity (30%). The reason for this choice may also be related to the age of the respondents (64.2 ± 9.7 years). As it is well known, increasing physical activity and weight reduction in people over 60 is problematic, which may be due to the lack of knowledge, comorbidities that negatively affect physical fitness, or quick discouragement due to the lack of visible results adequate to the effort put in by the patient.

Another problem that already reaches the scale of the epidemic is overweight and obesity. Excessive body weight is the main cause of hypertension, type 2 diabetes, and thus consequently leads to the development of cardiovascular diseases. According to studies conducted in Europe and the USA, overweight and obesity tend to increase year by year [12, 25]. Similarly, in Poland, a significant part of the population has problems maintaining a healthy body weight. The data of the Central Statistical Office show that over 18 years, the number of overweight people increased more than 2 times (F: 14.2% vs. 30.1; M: 18.7 vs. 44.1%), and with obesity, respectively for women 12.4% vs. 15.6% and for men 10.3% vs. 18.1% [26]. This trend continues across Europe, where obesity has increased from 32 to 39% [12, 27]. These results are similar to those obtained in our study, in which 44% and 35% of the respondents were overweight and obese, respectively. Additionally, abdominal obesity was observed in 52% of men (waist circumference > 102 cm) and as many as 76% of women (waist circumference > 88 cm). The epidemic of overweight and obesity is associated with an increased incidence of type 2 diabetes [28]. In the studied group, this disease occurred in 32% of people, which is comparable with its prevalence in people with coronary heart disease (CHD) in the European population (27%) [12, 27].

The Norwegian Myocardial Infarction Register showed that only 1% of patients after myocardial infarction (MI) reached all secondary prevention treatment targets. Patients with CAD hospitalized with type 1 myocardial infarction in Norway from 2013 to 2016 reached LDL < 1.8 mmol/L (< 70 mg/dL) in 25% and blood pressure below 140/90 mmHg in 43%. Moreover, the study showed that MI occurs in as many as 25% of patients with prior CAD [29]. In the Tromsø 7 study (2015–2016) among 1483 participants with CHD (50.8% with previous MI, 31.4% with angina pectoris, and 82.7% with previous PCI and/or CABG) target LDL levels were reached in 9% of the subjects, and blood pressure < 140/90 mmHg in 58%. The prevalence of pharmacological CHD treatment was 76% for lipid-lowering drugs (LLDs) and 72% for antihypertensive drugs. There was only a strong association between using LLDs and achieving the treatment goal for LDL cholesterol [30]. Kaldal et al. investigated the effects of a long-term hospital-based secondary prevention follow-up program in patients aged 18–80 years and admitted to the hospital with a diagnosis of MI or after scheduled PCI/CABG. Patients were randomized to hospital-based secondary preventive care with consultations at 2 weeks, 3 months, 6 months, and 1 year after the index event and annually for up to 5 years, or to primary care follow-up. Final data was collected after 10 years. The LDL cholesterol levels were significantly lower in the intervention arm of the study (hospital-based follow-up), but the effect disappeared after the cessation of annual consultations. After the first year, target LDL levels were reached by 79% of patients from hospital-based follow-up, while after 10 years it was only 43%. During the first year, most patients took only statins as a cholesterol-lowering drug, and then in subsequent years of the follow-up, ezetimibe was added to the treatment, but proprotein convertase subtilisin/kexin type 9 (PCSK9) inhibitors were not used. After the first year, target blood pressure < 140/90 mmHg was reached by 68% of patients from hospital-based follow-up, while after 10 years it was only 53% [31]. In our study, only 2% of the participants achieved LDL < 55 mg/dL, and 7% reached LDL < 70 mg/dL. Blood pressure < 140/90 mmHg was observed in 77%. Patients were not taking PCSK9 inhibitors or ezetimibe. In the majority of patients, LDL levels were not controlled with adequate frequency due to a lack of patient knowledge and physician consent. Patients were not monitored so intensively during the follow-up period, which averaged 3 years and 8 months after the first coronary intervention. In contrast, in the EUROASPIRE V study, LDL level < 1.8 mmol/L (< 70 mg/dL) was achieved by about 30% of the subjects and PCSK9 inhibitors were used in only 15 patients out of 7824 and only 2.7% of the participants were on a combination of a high-intensity statin with ezetimibe at an interview after hospitalization for a coronary event. In DYSIS II study, 23.8% of participants with ACS and 23.2% with stable CHD achieved LDL < 1.8 mmol/L (< 70 mg/dL) using LLT (79.8% were receiving statin monotherapy and 11.6% a combination of statin plus ezetimibe). However, in the absence of LLT, only 3.6% of subjects with ACS and 2.9% with stable CHD reached LDL < 1.8 mmol/L (< 70 mg/dL) [21]. In the DAVINCI study, adding PCSK9 inhibitors to ezetimibe and a statin in patients with ASCVD, LDL < 1.8 mmol/L (< 70 mg/dL) was achieved in 67% and LDL < 1.4 mmol/L (< 55 mg/dL) in 58% [22]. This leaves room for further contributions to the secondary prevention of CVD if the availability of PCSK9 inhibitors and ezetimibe increases, and preventive care with frequent consultations as well as the awareness of patients and physicians about the appropriate frequency of cholesterol control improves.

Failure to implement the recommendations and appropriate control of risk factors for cardiovascular diseases in our patients could have a significant impact on the progression of CAD, which resulted in the fact that almost half of the patients underwent repeated revascularization procedures. Coronary angioplasty was performed in 40% of the patients and 8% of the patients were qualified for cardiac surgery of CABG. The premises resulting from the WOBASZ study [17] or the Polish part of the EUROASPIRE study [23], as well as from this study, should have a significant impact on undertaking a corrective process, which may in the near future reduce cardiovascular mortality. The corrective process should include educating the patient and their families in order to modify risk factors, conducting rational pharmacotherapy, as well as routine specialist checkups, which should result in increased patient mobilization in the therapeutic process [16, 18]. Modification of the current lifestyle is a key element in the process of treatment and prevention of further serious cardiovascular consequences such as another heart attack, stroke, or even death. The key issue in the entire treatment process is the consistent building of positive relationships with the patient, and routine checkups with general practitioners and specialists at an appropriate frequency. This will allow for quick and easy detection of irregularities as well as their modification, thus preventing serious consequences in the future. In the effective prevention of cardiovascular diseases, it is extremely important to consistently educate the society in this field. The continuation of epidemiological research is the foundation that will help to find the causes of therapy failure, which will allow for the implementation of appropriate corrective measures.

## Conclusions

Based on the analysis of the study group, the following conclusions were obtained:

1. Less than half of the people after coronary interventions undertook pro-health behaviors that required personal effort and resignation from previous habits, such as quitting smoking, reducing alcohol consumption, reducing body weight or increasing physical activity;
2. younger people significantly more often increased physical activity, limited alcohol consumption, and quit smoking compared to older people;
3. men significantly more often than women limited their alcohol consumption;
4. people with secondary and higher education significantly more often increased their physical activity and reduced alcohol consumption compared to people with lower education;
5. people with past acute coronary syndrome were significantly more likely to quit smoking and reduce alcohol consumption compared to patients without a history of acute coronary syndrome;
6. the vast majority of patients after coronary surgery did not achieve the recommended levels of LDL cholesterol and triglycerides in the treatment, consistent with the ESC guidelines in the secondary prevention of coronary disease;
7. there is a need for better cooperation in the modification of risk factors and treatment of CHD, both on the part of the patient and the physician;
8. inadequate control of risk factors may result in faster disease progression and the need for coronary re-interventions;
9. the current system and implementation of secondary prevention of cardiovascular diseases seem insufficient and ineffective.

## Data Availability

All of the data are stored digitally by the researchers.
